# Electrocatalytic oxidation of pyrrole on a quasi‐reversible silver nanodumbbell particle surface for supramolecular porphyrin production

**DOI:** 10.1002/open.202300212

**Published:** 2024-02-13

**Authors:** Olayemi Jola Fakayode, Reagan L. Mohlala, Rudzani Ratshiedana, Bambesiwe M. May, Eno E. Ebenso, Usisipho Feleni, Thabo T.I. Nkambule

**Affiliations:** ^1^ Institute for Nanotechnology and Water Sustainability (iNanoWS) College of Science Engineering and Technology University of South Africa, Florida Campus, 28 Pioneer Avenue, Roodepoort 1709 Johannesburg South Africa; ^2^ Advanced Material Science Division, Mintek 200 Malibongwe Drive, Randburg Johannesburg South Africa; ^3^ Centre for Materials Science College of Science Engineering and Technology University of South Africa, Florida Campus 28 Pioneer Avenue, Roodepoort 1709 Johannesburg South Africa

**Keywords:** electro-synthesis, porphyrin, pyrrole, light-harvesting, photophysical

## Abstract

Photoactive supramolecular porphyrin assemblies are attractive molecules for light‐harvesting applications. This is due to their relatively non‐toxicity, biological activities and charge and energy exchange characteristics. However, the extreme cost associated with their synthesis and requirements for toxic organic solvents during purification pose a challenge to the sustainability characteristics of their applications. This work presents the first report on the sustainable synthesis, spectroscopic and photophysical characterizations of a near‐infrared (NIR) absorbing Ca(II)‐meso‐tetrakis (4‐hydroxyphenyl)porphyrin using an electrolyzed pyrrole solution. The latter was obtained by cycling the pyrrole solution across the silver nanodumbbell particle surface at room temperature. The electrolyzed solution condensed readily with acidified p‐hydroxybenzaldehyde, producing the targeted purple porphyrin. The non‐electrolyzed pyrrole solution formed a green substance with significantly different optical properties. Remarkable differences were observed in the voltammograms of the silver nanodumbbell particles and those of the conventional gold electrode during the pyrrole cycling, suggesting different routes of porphyrin formation. The rationale behind these formations and the associated mechanisms were extensively discussed. Metalation with aqueous Ca^2+^ ion caused a Stokes shift of 38.75 eV. The current study shows the advantage of the electrochemical method towards obtaining sustainable light‐harvesting porphyrin at room temperature without the need for high‐energy‐dependent conventional processes.

## Graphical abstract

This work reports the synthesis of meso‐tetrakis(4‐hydroxyphenyl)porphyrin through condensation of an electrolyzed pyrrole solution with an acidified 4‐hydroxybenzaldehyde at room temperature. It describes the use of silver nanodumbbell particles as sustainable electrode materials for symmetric porphyrin production. Metalation and optical characterizations together with various techniques such as ^1^HNMR and ^13^CNMR confirm the synthesis of the targeted product.

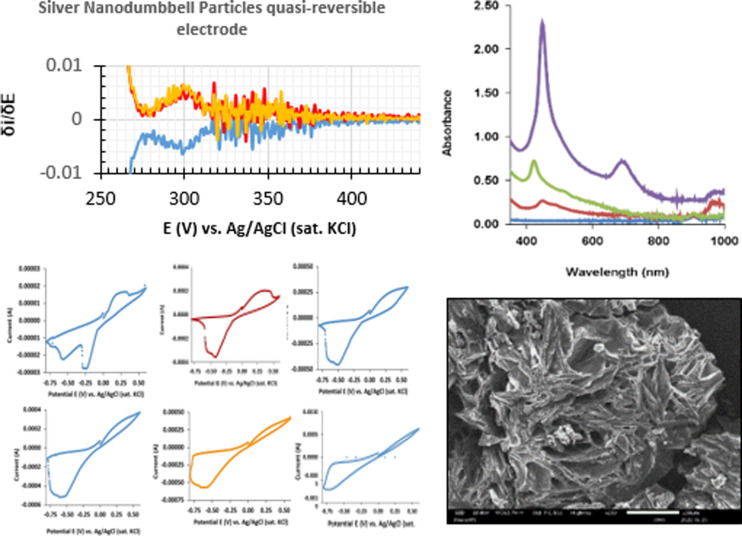

## Introduction

Porphyrins are remarkable molecules that have attracted applications in various fields, including cancer and bacterial infection treatments,[^1^, ^2^, ^3^, ^4^] solar energy harvesting, and pollutant detection. Metalloporphyrin complexes, in particular, have the potential to revolutionize the energy industry with their charge and photon exchange capabilities.[^5^, ^6^, ^7^] With the discovery of porphyrins in natural sources, synthetic porphyrins are now being created that can be used in biomedical imaging, organic synthesis, sensing,[^8^, ^9^] and water treatment. By tuning these molecules through oxidation, reduction, protonation, and substitution of their meso‐ and core sites, they can be channelled to perform better functions. Adding 4‐hydroxyphenyl units to the meso positions of porphyrin is just one example of how these molecules can be customized to suit specific needs. This simple modification can shift the Qx (0,0) band to a higher wavelength, making it useful in light‐harvesting cancer treatment and solar energy‐based technology applications. With such versatility, porphyrins are projected to inspire more innovations and progress in science and technology over time.

The condensation of pyrrole with an organic aldehyde in an acidic medium usually affords porphyrins. This process has been employed under different strategies such as direct classical reflux, 2‐step porphyrinogen formation and oxidation, microwave‐assisted synthesis and open‐chain tetrapyrrole electrochemical synthesis. However, the generation of impurities and the requirement for tedious purifications challenge the sustainability of these practices. Consequently, new approaches for porphyrin synthesis are continuously being researched and developed.

The evolution of green chemistry has paved the way to redesigning and restructuring the pathway to porphyrin synthesis. For example, an attempt to synthesize porphyrins directly in aqueous solutions has been achieved. Pyrrole is soluble in water. As a result, condensation with acidified aldehyde in aqueous environment can be hypothesized and investigated.

Electrochemical synthesis exhibits some advantages over conventional practices. These include synthesis under a controlled atmosphere, reliable repeatability, low material requirement and lower costs. Also, undesired pollutants such as CO_2_,^[10]^ CO and NH_3_, which sometimes appear during synthesis, can be separated from the targeted products or reconverted to non‐interfering products. However, despite these advantages, many electrodes commonly used for organic compound oxidation exhibit too fast electron exchange properties, causing a lot of impurities in the precursor solution or are too expensive to acquire or maintain. Thus, sustainable electrodes are currently being researched and developed. One resolution to this challenge is to employ transition metal‐based electrodes with quasi‐reversible or irreversible characteristics. This reversibility properties will ensure a controlled oxidation process (since the electron transfer process is moderate or very slow). This will help avoid the generation of multiple impurities.

Quasi‐reversibility of an electrode occurs when the difference between the oxidation and reduction peak potentials of a redox couple is greater than 57 mV/n at 25 °C (298 K) (n being the no. of the exchanged electron). In other words, for n=1, an electrode shows quasi‐reversibility behavior when ▴Ep>57 mV at 25 °C (298 K).[^11^, ^12^, ^13^] In addition, an electrode can exhibit quasi‐reversibility when the ratio of the faradaic oxidation current to that of the reduction current deviates from unity, i. e. I_anode_/I_cathode_ < or > 1.^[14]^ In their works, on differentiating quasi‐reversibility from reversibility and irreversibility, Birke and coworkers use the following parameters, emphasizing the importance of charge transfer coefficient, α:^[14]^ reversible electron exchange: Ipa/Ipc=1, quasi reversible electron exchange: lpa/Ipc<1 for α>or=0.5 or lpa/Ipc < or > 1 for α<0.5 and irreversible electron exchange: lpa/Ipc<1. In contrast to the reversible counterpart, quasi‐reversibility refers to a slower electron transfer process^[15]^ while irreversibility implies extremely slow electron exchange.

Many previous reports showed the utility of quasi‐reversible electrodes for various functions. For example, Obuah and coworkers reported using a quasi‐reversible glassy carbon electrode (GCE) for the electrocatalytic conversion of ethylene to 1‐butene and associated oligomers.^[11]^ Also, Lui et al. used a quasi‐reversible GCE to determine dopamine in an injectable medium.^[16]^

Silver nanoparticles are materials used for various functions, such as sensing^[17]^ and catalysis. They exhibit different morphologies, including spheres,^[18]^ rods, cubes, prisms and dumbbells.^[19]^ The functionality of silver nanoparticles depends on their morphologies. For example, Helmlinger and coworkers reported the effect of the size and shape of silver nanoparticles on cytotoxicity and bacterial activities.^[20]^ Also, Bansal et al., in their report, showed the effects of electrocatalytic properties of silver nanoparticles based on morphologies on oxidation of hydrazine and formaldehyde and reduction of hydrogen peroxide.^[21]^

Localized surface plasmon resonance (LSPR) is a specialized optical property shown by noble metals such as Ag and Au. They occur when the free electrons on the surface of these metals interact with lights of suitable wavelengths. Gold shows an SPR peak around 520 nm. The characteristic SPR of silver usually peaks around 420 nm. The SPR properties are usually probed using ultraviolet‐visible spectrophotometers based on the principle of light absorption. Noble metal SPR properties are employed for applications such as sensing[^22^, ^23^] and catalysis.^[24]^

This paper reports the electrochemical cycling of the pyrrole solution for the production of a targeted porphyrin (meso‐tetrakis(4‐hydroxyphenyl)porphyrin at room temperature using a hydrophobic silver nanodumbbell electrocatalyst particle electrode. The cycling of the solution across silver nanodumbbell particles’ surface and condensation of the resulting solution with acidified 4‐hydroxybenzaldehyde afforded the targeted porphyrin without heating, distillation, or purification using organic solvents. Conversion of the free base planarity to the D_4h_ symmetry was achieved by interaction with Ca^2+^ ions. The as‐synthesized porphyrin was recrystallized in ethanol to obtain a dark purple material. The yield was about 20.75 %. The control solution, i. e. a mixture of the non‐electrolyzed pyrrole and acidified 4‐hydroxybenzaldehyde, exhibited a green colour prior to and after metalation with Ca (II) ions, suggesting the formation of a different material (the as‐synthesized porphyrin showed dark purple colour prior to and green colour after metalation with Ca (II) ions).

## Results and Discussion

### Electrode Surface Characterization

**Figure 1:**
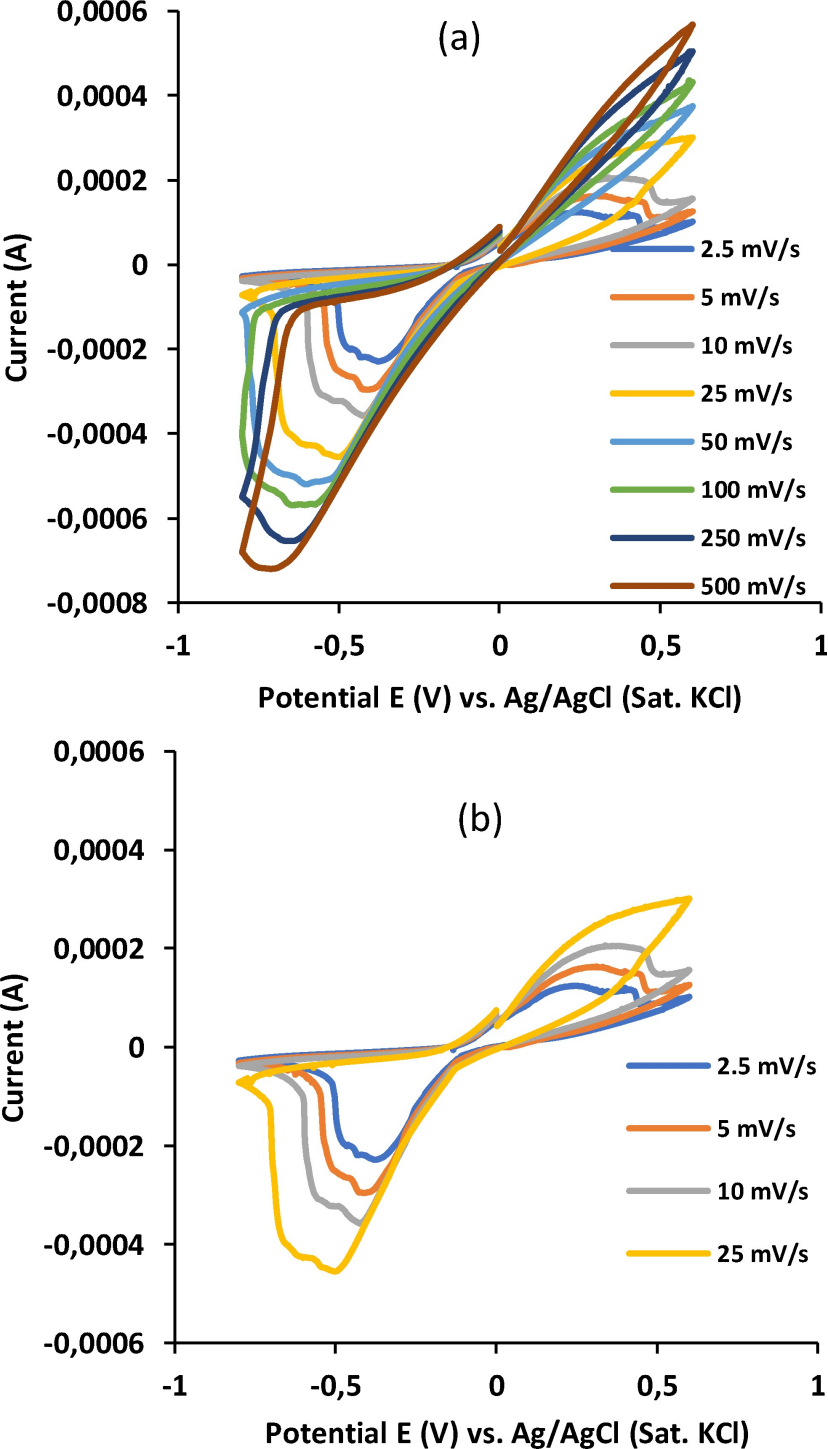
Voltammograms at different scan rates in the presence of ferro‐cyanide redox probe. (a) 1–500 mV/s; (b) 2.5–25 mV/s. Reference electrode: Ag/AgCl (sat. KCl).

The results of the cyclic voltammetric evaluation of the electrocatalyst's surface in the presence of ferrocyanide redox probe are given in Figure 1a–i. As shown in Figure 1a‐i, well‐defined quasi‐reversible voltammograms, the currents of which increased with increasing scan rates (1 to 25 mV/s), were observed between the potential range of −1 to +1 V. This implied that the anodic and the cathodic reactions at these scan rates were diffusion‐controlled.

However, as the scan rate increased from 25–500 mV/s, deviation from the linearity occurred, probably due to surface adsorption. This deviation affects the oxidation current at the anode, causing it to decrease (closing‐ups of the anodic signals as the scan rates increase). In contrast, the current at the cathode increased with the scan rate, causing more diffusion at the cathode than at the anode. Moreover, due to the small dimension of the exposed electrocatalyst's surface (tip diameter – 0.80 mm), a small number of molecules would adsorb on its surface, forcing the remaining molecules to remain in solution. This explained the higher cathodic signals observed in the voltammograms at all scan rates compared to those at the anode. This effect was clearly seen in the first derivative plots (Fig. S1a–i, Supplementary Material). However, as shown in Fig. S1a–i (Supplementary Material), the dependence of the cathodic peak on the scan rate increased from 2.5 to 100 mV/s, after which the relationship became inverse.

**Figure 2:**
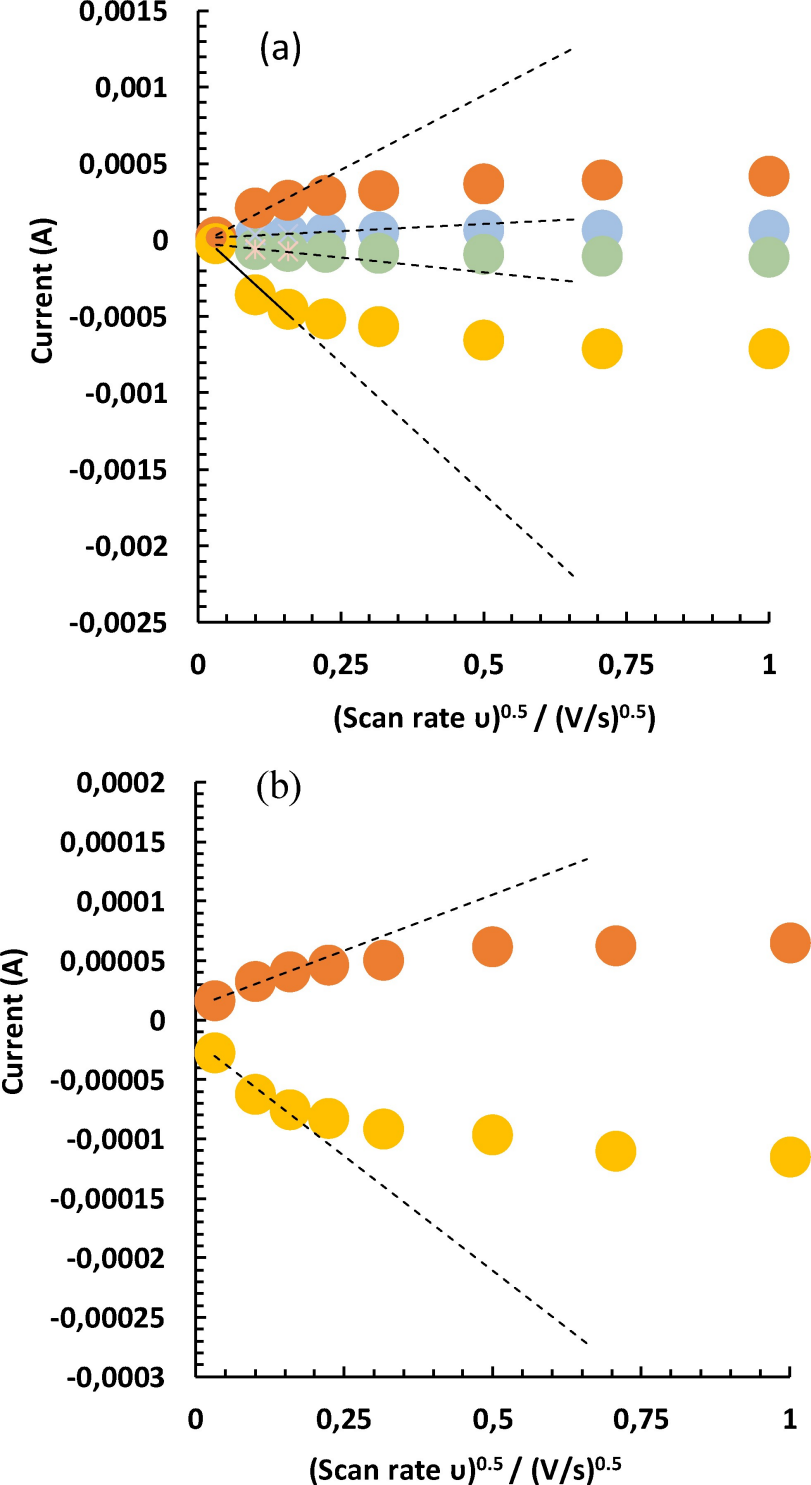
Reversibility plots of current vs square root of the scan rates in the presence of the ferro‐cyanide probe. (a) Combined plots: A1 (First anode data), C1 (First cathode data), A2 (Second anode data), C2 (Second cathode data), n=2; (b) Enlargement of A1 and C1 in (a). Scan rates: 1.0–1000 mV/s. Reference electrode: Ag/AgCl (sat. KCl).

The results of the reversibility plots of the interaction of the cyanide probe on the silver nanodumbbell particle electrode are shown in Figure 2and Table 1.

**Table 1:** Reversibility data of the ferro‐cyanide probe on silver nanodumbbell particle surface^a^.

υ (mV/s)	Epa (V)	Epc (V)	▴Epa,c = Epa‐Epc (V)	▴Epa,c (mV)	Ipa (μA)	Ipc (μA)	Ipa/Ipc
1	0.25	−0.26	0.51	510	16.3	−27.5	−0.594
10	0.30	−0.38	0.68	680	32.3	−62.3	−0.518
25	0.35	−0.55	0.9	900	40.0	−75.8	−0.527
50	0.35	−0.55	0.9	900	46.0	−83.2	−0.554
100	0.35	−0.60	0.95	950	50.1	−92.0	−0.544
250	0.35	−0.62	0.97	970	61.4	−96.6	−0.636
500	0.35	−0.66	1.01	1010	62.3	−110.5	−0.564
1000	0.35	−0.66	1.01	1010	64.4	−115.3	−0.558
(Repeat Data)							

υ (mV/s)	Epa	Epc	▴Epa,c=Epa‐Epc (V)	▴Epa,c (mV)	Ipa (μA)	Ipc x (μA)	Ipa/Ipc
1	0.200	−0.25	0.450	450	15.6	−27.4	−0.568
10	0.350	−0.425	0.775	775	204.4	−357.6	−0.571
25	0.350	−0.500	0.850	850	259.5	−454.9	−0.570
50	0.350	−0.600	0.950	950	290.8	−519.4	−0.560
100	0.350	−0.650	1.000	1000	318.0	−568.5	−0.559
250	0.350	−0.650	1.000	1000	362.5	−654.0	−0.554
500	0.350	−0.750	1.100	1100	390.0	−716.6	−0.544
1000	0.350	−0.750	1.100	1100	413.0	−713.2	−0.579

^a^Temperature=24 °C.

As shown in Figure 2 and Table 1, the nanoparticle electrode exhibited quasi‐reversible characteristics since its change in the peak potentials was higher than 54.72 mV expected for a reversible condition at 24 °C, for a one‐electron exchange redox reaction (57 mV for 25 °C).[^11^, ^12^, ^13^]

In addition, the ratio of the peak currents Ipa/Ipc was less than one, providing evidence of deviation from the reversible behaviour.^[14]^ Evidence of diffusion‐controlled process can be seen on both anodic and cathodic sites, majorly between 10–50 mV/s (R^2^=0.9766, 0.934 and R^2^=0.9594, 0.9349 for anodic and cathodic processes respectively; n=2).

**Scheme 1:**
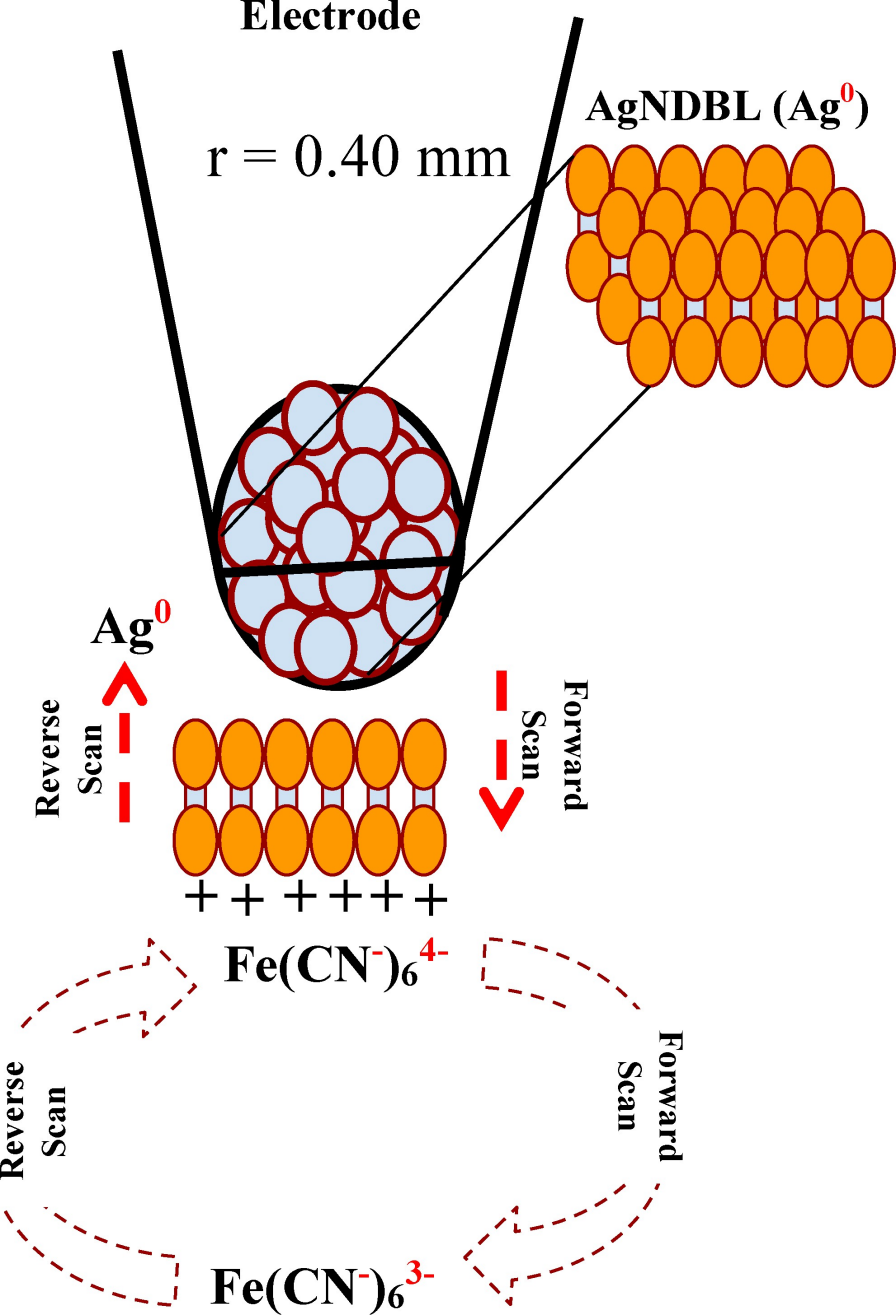
Illustration of the interaction of ferro‐cyanide at the surface of the silver nanodumbbell (Ag‐NDBL) electrode.

The complexity associated with the anodic process at higher scan rates may be explained based on the fact that the positively charged Ag^+^ ion released during the forward scan could electrostatically bind to the negatively charged ferrocyanide, causing temporary adsorption to take place at the surface of the particulate electrode (Scheme 1).

**Figure 3:**
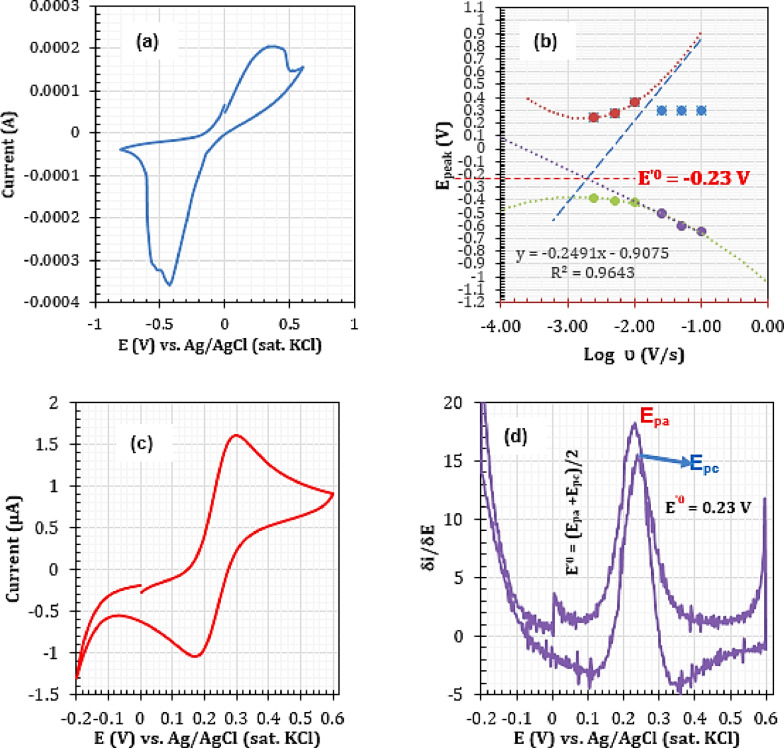
Surface redox voltammetric characteristics of the Ag‐NDBL electrocatalyst. (a) CV of AgNDBL at 10 mV/s; (b) Laviron's plots of AgNDBL at 10 mV/s; (c) Comparative voltammogram of a conventional gold‐plated electrode (1.5 mm diameter, scan rate=10 mV/s)); (d) First derivative plots of CV of Au electrode at 10 mV/s. Redox probe: Fe(CN)64‐/KCl (0.1 M). Temperature: Au: 295 K, AgNDBL: 297 K.

Under a typical electrochemical process, the evolution of chlorine gas may be expected within the range of the scans employed in this study. However, this gaseous release depends on the concentration of the chloride ion in the aqueous solution and the type of electrode used (Electrolysis of brine vs. dilute NaCl). Under the current electrochemical process, no chlorine evolution was detected. This might be due to the low concentration of chloride ions in the solution or unfavourable conditions on the electrode surface due to the oxidation of the silver nanodumbbell particles. As shown in Figure 3a–b, a formal surface standard reduction potential of 0.23 was obtained for the ferro/ferri‐cyanide couple on the quasi‐reversible Ag‐NDBL surface. This value agreed very well with that obtained using the conventional reversible Au electrode (Figure 3a–d). The electrochemical rate constant was in the order 10^−3^ s‐1, which showed a feasible charge transfer occurrence (transfer coefficient α=0.23) (Table 2).

**Table 2:** Electrochemical kinetics data of silver nanodumbbell powder electrode*.

αc=−2.3 RT/Sc(nF)	αa=1‐αc	Sc	Electrochemical rate constant k_s_ based on E_P_ vs. Log υ (s^−1^)
0.23	0.77	−0.2491	1×10^−3^

*αc=transfer coefficient (cathode); αa=transfer coefficient (anode); ks=electrochemical rate constant; Sc=slope of the plot of E_peak_ vs, Log υ for the cathode reaction. υ=scan rate.

The lower value of α (α <0.5) confirmed the quasi‐reversible nature of the electrode process.^[14]^ According to Laviron, the most important parameter that determines the irreversibility behaviour of a redox species on an electrode's surface is the difference in the anodic and cathodic peak potentials (ΔEp(a–c)). Plotting Ep(a,c) against the Log of scan rate can provide valuable information such as electrochemical rate constant ks and charge transfer coefficient α.^[14]^ According to Laviron, provided nΔEp(a–c) >200 mV, ΔEp(a‐c) relates to ks as follows (Eq. 1:

(1) Logks=αlog(1-α)+(1-α)logα-log(RT/nFυ)-α(1-α)nF(ΔEp(a-c))/2.3RT

Where α=Cathodic transfer coefficient=αc

(2) αc=-2.3RT/slope(nF)

Epc=Cathodic peak potential, υ=Scan rate, R=Universal gas constant=8.314 kJ/mol, n=no. of electron, F=Faraday's constant=96485 sA/mol, T=Temperature in Kelvin=295 K in this study.

Thus, from Eq. 2, a plot of Ep(c) vs. log υ gave a straight line whose slope equalled −0.2491 (Figure 33a–b, Table 1). As a result, a cathodic transfer coefficient (αc) of 0.23 was estimated using the relation in Eq. 2.

Rearranging Eq. 1 led to

(3) α(1-α)nF(ΔEp(a-c))/2.3RT=αlog(1-α)+(1-α)logα-Logks-log(RT/nFυ)

Thus, a plot of α(1‐ α)nF/2.3 RT (ΔEp(a‐c)) vs. log RT/nFυ should give a negative slope straight line whose intercept Ї equals to:
(4)

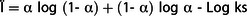




Thus,
(5)

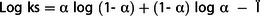



(6)

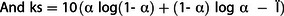




Alternatively, ks may be estimated from:

(7) ks=αcnFυc/RT

υc being equal to the scan rate at the point when the extrapolating slope line reaches “Ep(c)=0” from the plots of Ep(c) vs. Log scan rate (Figure 3a–b).

The results of the estimation of the electrochemical rate constant using the latter method are given in Table 2. As shown in Table 2, the values of k_s_ agreed very well with the reversibility nature of the electrode (Figure 3a–d).

### Oxidation of Pyrrole and Porphyrin Synthesis

**Figure 4:**
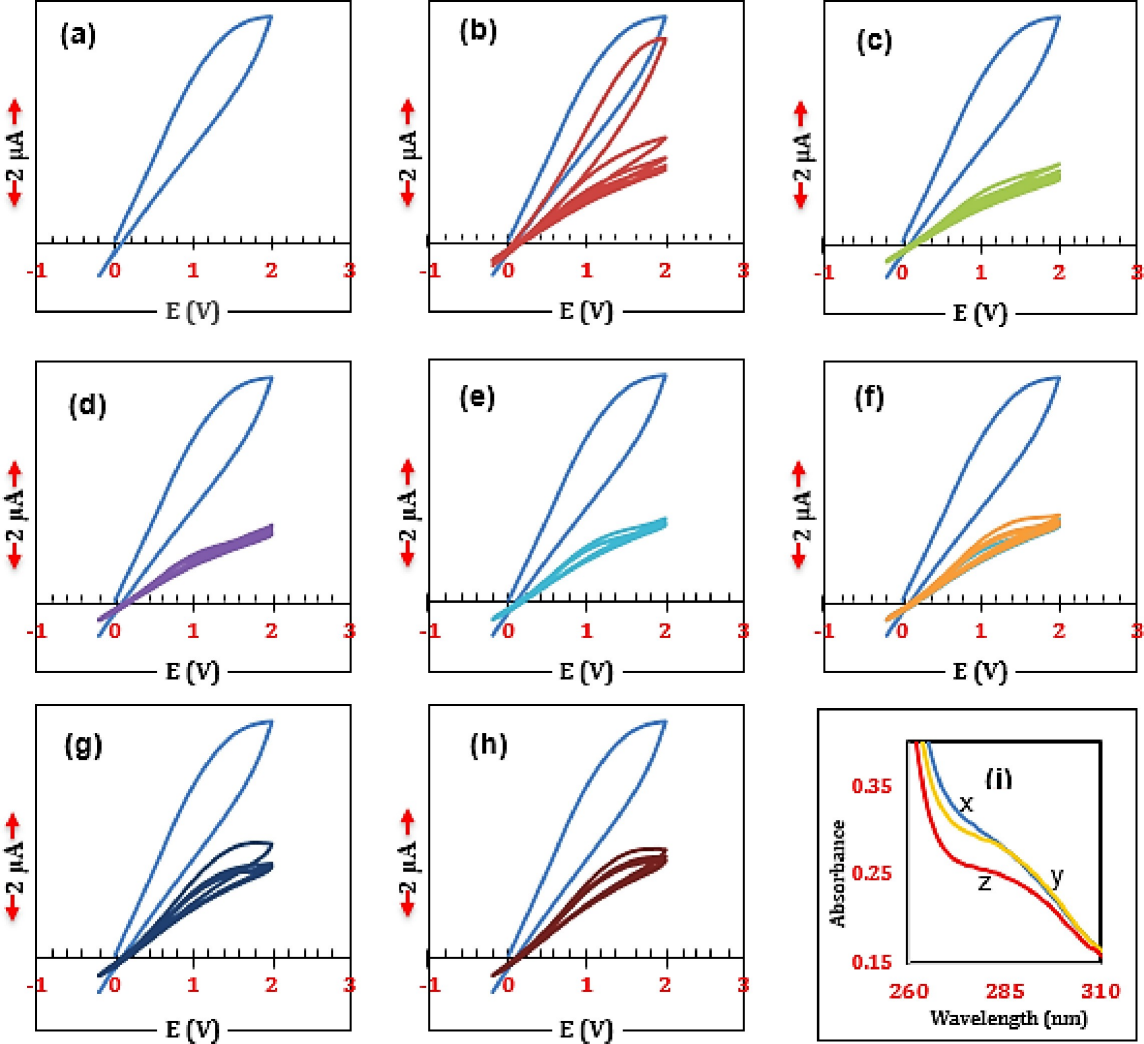
Electrocatalytic oxidation of pyrrole on Ag‐NDBL surface at different voltammetric cycles. (a) Blank's CV; (b) Blank, 1–5 cycles; (c) Blank, 6–10 cycles; (d) Blank, 11–15 cycles; (e) Blank, 16–20; (f) Blank, 21–25 cycles; (g) Blank, 26–30 cycles; (h) ) Blank, 31–35 cycles; (i) UV‐Vis of the pyrrole solution before (x), after 35th cycling on AgNDBL (y) and after 35th cycling on conventional Au (z) electrode surfaces. Blank: 0.1 M KCl. Scan rate 25 mV/s (Technique: Cyclic voltammetry). Reference electrode: Ag/AgCl (sat. KCl).

The results of the electro‐oxidation of pyrrole in KCl solution are given in Figure 4a–h. As shown in Figure 4a–h, the voltammograms exhibited a decrease in the anodic current as the number of cycles increased. This can be attributed to the competitive presence of chloride ions on the electrocatalyst's surface. Chloride ions are known to form complexes with noble metal ions. Thus, Ag‐chloride complex formation may occur at the surface of the electrode through the interaction of Cl^−^ and Ag^+^ ions (released during the anodic scan). Since no cathodic peak appeared during the reverse scan, it simply means the surface of the electrode would be covered with a large number of this species causing the current to decrease during subsequent scans. However, the presence of this complex could promote feasible oxidation of some of the pyrrole near the electrode surface through binding to the Ag^+^ ion in the AgCl^−^ complex, subjecting the molecule to adsorption and subsequently product desorption over time.

The evidence of this oxidation can be seen on the UV‐Vis spectra of the pyrrole solution after cycling (Figure 4i). As shown in Figure 4i, the absorbance of the pyrrole solution decreased after cycling using either the AgNDBL or a conventional Au electrode, the effect being greater using the latter. The greater depletion of the pyrrole solution using the Au electrode was due to the relatively faster electron kinetics on its surface (Figure 3a–d). This implied more oxidized species might be produced on this surface than on the AgNDBL electrode's surface. This act can make or mar the synthesis objective depending on the nature of the targeted molecules, whether free base or oxidized form. Negatively, it may introduce a lot of impurities, thus, incurring more cost to the synthesis process.

### Optical Characterization of the as‐Synthesized Porphyrin and its Ca(II)‐complex

**Figure 5:**
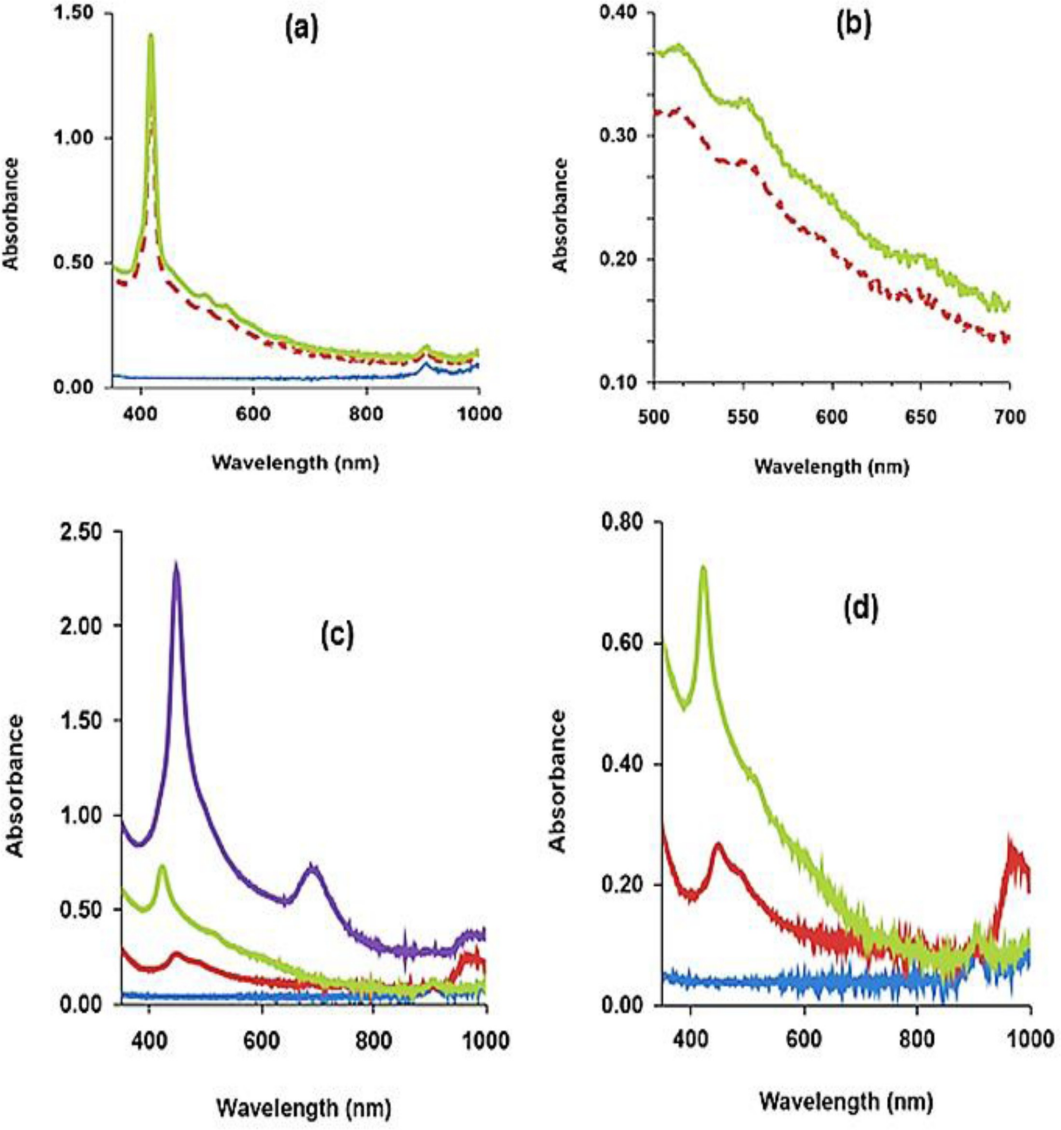
UV‐Vis of the porphyrin synthesized using the electrolyzed pyrrole solution cycled on AgNDBL surface (a) Free base (D_2h_) symmetric porphyrin showing the strongly intense Soret band and weakly intense Q bands(Green (concentrated) or red dashed (diluted) line (blue line=ethanol (blank); (b) Enlargement of the Q bands in (a); (c) Ca(II)‐porphyrin complex(upper spectrum) (D_4h_ symmetry) (upper spectrum) and precipitated porphyrin after solvent evaporation (6 days aging in the air) (middle spectrum), supernatant lower spectrum (6 days aging in the air) and blank (ethanol, lowest spectrum) (blue line); (d) Enlargement of the lower spectra in (c).

After the 35th cycle, the electrolyzed pyrrole solution condensed with 4‐hydroxybenzaldehyde to form a purple porphyrin (Figure 5a–b). As shown in Figure 5a–b, well‐defined Soret band at 419 nm and Q bands at 517, 555, 600 and 655 were associated with the as‐synthesized porphyrin(meso‐tetrakis(4‐hydroxyphenyl)porphyrin).[^25^, ^26^] The peak at around 900 nm was due to water absorption.

In contrast to the conventional practices, this synthesis did not involve redistillation of the pyrrole solution or heating/refluxing, suggesting its cost‐advantage attribute. Metallation with Ca (II) caused a distortion in the planarity of the free base as expected, forming a green complex. Figure 5c shows the absorption spectrum of this complex. As shown in Figure 5c, three‐band peaks, viz: a Soret at 448 nm and two NIR Q‐bands at 698 and 980 nm, characterized the complex (Table 3).

**Table 3:** Comparative absorption and emission data of the as‐synthesized porphyrin with the literature data.

Soret	QIV Qy 1,0	QIII Qy 0,0	QII Qx 1,0	QI Qx 0,0	MT	Sy	λ_e_ Q (0,0)	λ_Ph_	S_s_ (eV)	R_f_
419	517	*555*	600	655	CV^a^	D_2h_	659	730	309.96	TW
448	–	–	–	698,980	CV^a^/ met	D_4h_	–	730	38.75	TW
426	520	560	600	653	CS^b^	D_2h_	656	x	413.28	[25]
426	530	566	609	656	CS^b^	D_2h_	650		206.64	[26]
419 ^*^	x	x	x	646	CS^b^	D_2h_	652	x	206.64	[27]
447	X	X	x	688	CS^b^	D_4h_	712	x	51.66	[28]
420	515	555	580	650	CV^c^	D_2h_	655	730	247.97	[29]
450	‐	‐	‐	695	CV^c^ / met	D_4h_	x	x	x	[29]

^a^ by silver nanodumbbell particles ^b^conventional wet chemical approach involving refluxing and purification by organic solvents; ^c^ by gold electrode; *=for tetraphenyporphyrin; ‐=absent; x=undisclosed; CS=Classical; CV=Cyclic voltammetry; met=metalation; MT=Method; Sy=Symmetry; S_s_ = Stokes Shift; λ_Ph_=Phosphorescence wavelength; λ_e_=Emission wavelength; TW=This work; R_f_=Reference. All values in nm except Stokes shifts (eV); E (eV)=1239.8 / λ (nm).

The red shifts of the Q‐band together with the fusion of the Q‐bands into two or single band is common for the porphyrins after metalation or protonation.[^25^, ^27^, ^28^, ^29^] However, the current study showed a near‐infrared Q‐band, in addition to the single Q‐band. After evaporation of the solvent (6 days in the presence of air), the complex precipitated as a brown solid. The absorption spectra of this precipitate (dissolved in ethanol) and its aged supernatant are shown in Figure 5c–d. As shown in Figure 5c–d, this evaporation‐induced self‐assemblies caused a transition from D_4h_ symmetry to the free base D_2h_ symmetry, suggesting the importance of stability by solvent in an aqueous solution.

**Figure 6:**
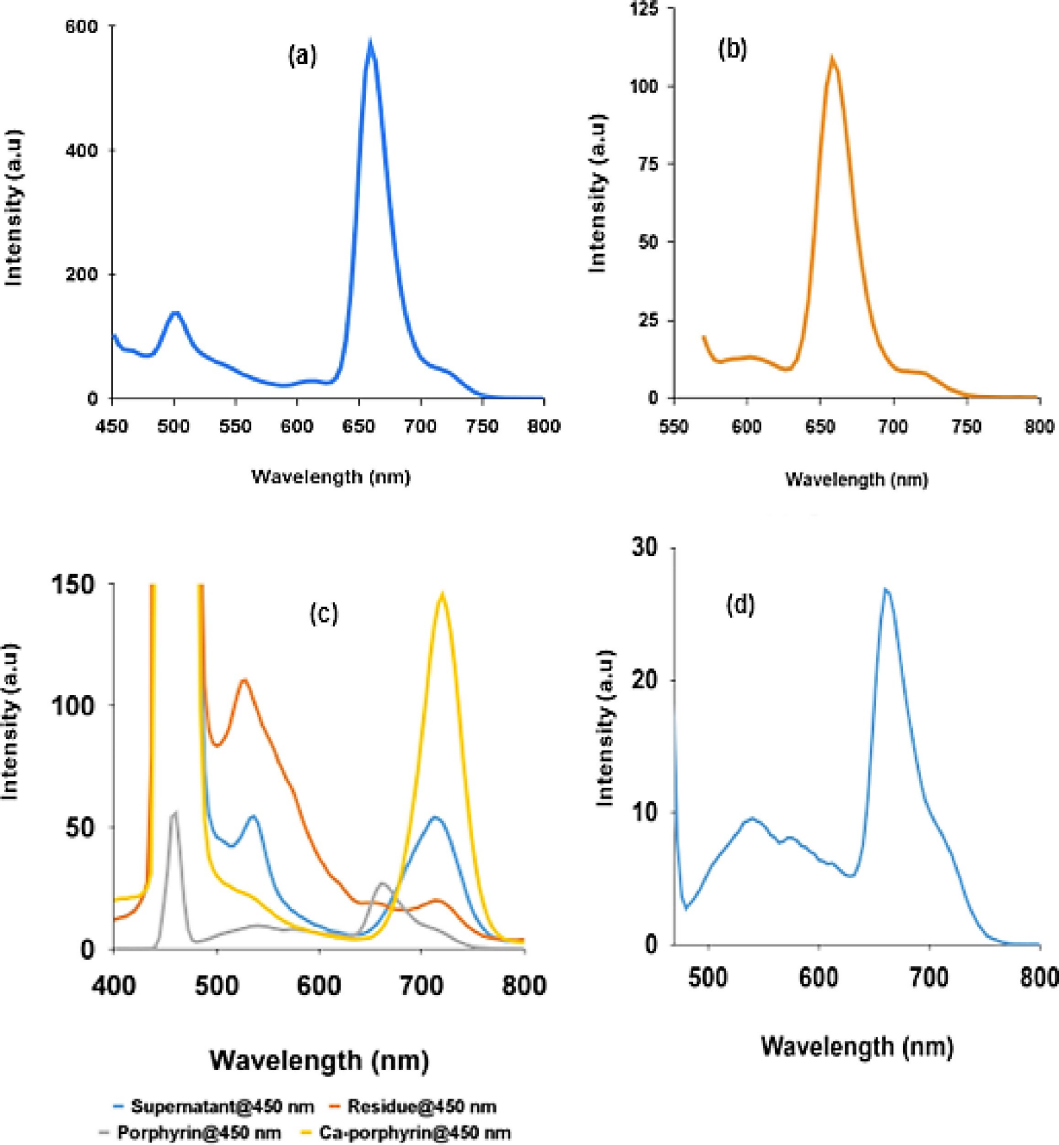
Photoluminescence (PL) spectra of the as‐synthesized porphyrin and its metallated calcium complex. (a) PL at 450 nm excitation (b) PL at 550 nm excitation; (c) PL of porphyrin, ca‐complex and precipitated form at 450 nm excitation; (d) Enlargement of the PL of the free base porphyrin in (c).

The results of the photoluminescence studies of the ethanolic solution of the as‐synthesized porphyrin and its ca‐complex are given in Figure 6a–d. As shown in Figure 6a–d, the as‐synthesized porphyrin exhibited an emission peak at 659 nm and a small phosphorescence peak at 730 nm under visible light excitations (450 and 550 nm) (Figure 6a–b).[^26^, ^27^] As revealed in Figure 6a–b, the emission band was a mirror image of the Q band (Q_x_ (0, 0)) and was independent of the excitation wavelength. After complexing with Ca^2+^ ion, the emission peak disappeared while the intensity of the phosphorescence increased dramatically (Figure 6c–d). However, after aging in the air for 6 days, the phosphorescence intensity decreased while that of the fluorescence increased supporting the transition from the metallated state to a free base state observed in the UV‐Vis spectra (Figure 5c–d and Figure 6c–d).

Proton nuclear magnetic resonance was further used to support the structure of the porphyrin. As shown in Table 4 and Fig. S2 (Supplementary Material), the porphyrin showed signals associated with meso‐substituted phenolic systems between 6.5–8.0 ppm, and free phenolic O−H between 9.50–10.0 ppm. Signals for β‐pyrrolic H between 8.0 −9.0 ppm and inner nitrogen proton which typically resonates between −2.5 and −3.0 ppm were absent. This might be due to the possibility of the formation of an N‐confused isomer of the targeted porphyrin (Chart 1) and solvent exchange effect on the inner core proton respectively. However, more work is currently ongoing to elucidate the reason behind these magnetic resonance displays. Another striking behaviour of the present porphyrin is the sharp difference in the ^1^H NMR data (obtained using the silver nanodumbbell particles) compared to those obtained using the gold‐plated electrode (previously reported by our team^[29]^ (synthesis under same conditions). This is despite the fact that these two porphyrins showed similar UV‐Vis spectra characteristics expected for the targeted meso‐tetrakis(4‐hydroxyphenyl)porphyrin (Figure 5a–b, Table 3). As shown in Table 4, the porphyrin produced using the Au electrode exhibited the usual inner core nitrogen‐H and β‐pyrrole proton at −2.87 and 8.89 ppm in DMSO‐d_6_ whereas the present porphyrin produced using the silver nanoparticles electrode showed absence of signals in these regions. The absence of signals in these regions might be due to solvent exchange and (or) the presence of N‐confused pyrrolic units as pointed out earlier.

**Table 4:** Comparative ^1^H NMR of the as‐synthesized porphyrin with reference compounds from the literature.

O‐H	Beta‐pyrrole H	Phenol‐ortho H	Phenol‐meta H	N‐H	Ref.
9.99 (s, 4H)	8.85 (s, 8H)	7.97 (d, 8H)	7.18 (d, 8H)	−2.92 (s, 2H)	[25]a
9.80 (s, 4H)	8.89 (s, 8H)	7.80 (d, 8H)	6.90 (d, 8H)	−2.87 (s, 2H)	[29]a
9.78 (s, 4H)	–	7.76 (d, 8H)	6.92 (d, 8H)	–	This work^a^
9.77 (s, 4H)	–	7.78 (d, 8H)	6.92 (d, 8H)	–	This work^b^

^a^Solvent: DMSO‐*d*_6_. ^b^Solvent: MeOD.

In addition, it was observed that after drying the ethanolic solutions of these porphyrins in laboratory‐based ceramic ware, the current porphyrin exhibited a stronger affinity to the components of the ceramic vessel than the one produced from the Au electrode. The stronger binding shown by the silver‐electrode‐produced porphyrin implies that this porphyrin may offer better dyeing potential than that formed from the Au electrode.

The results of the Carbon‐13 NMR of the as‐synthesized porphyrin are given in Table 5 and Fig. S3 (Supplementary Materials). As shown in Table 5 and Fig. S3 (Supplementary Materials), the α,β‐pyrrolic carbons together with the meso and phenyl carbons were detected at their expected chemical shifts, viz 155.5, 132,59, 120,5 and 100–125 ppm respectively (Chart 1).[^29^, ^30^] The 66.8 and 30.9 ppm signals were associated with deuterated methanol solvent.

**Table 5:** ^13^C NMR of the as‐synthesized porphyrin.

RC=O aldehyde	α‐pyrrole C	β‐pyrrole C	Meso‐C	Phenyl‐C	CH_2_ (solvent)	Solvent
Ppm						
		132.59	116.35	123.75	66.8	30.9^a^
183.5	155.5		120.5	107.25		30.9^b^

^a^Solvent: DMSO‐*d*_6_. ^b^Solvent: MeOD. TOC Text


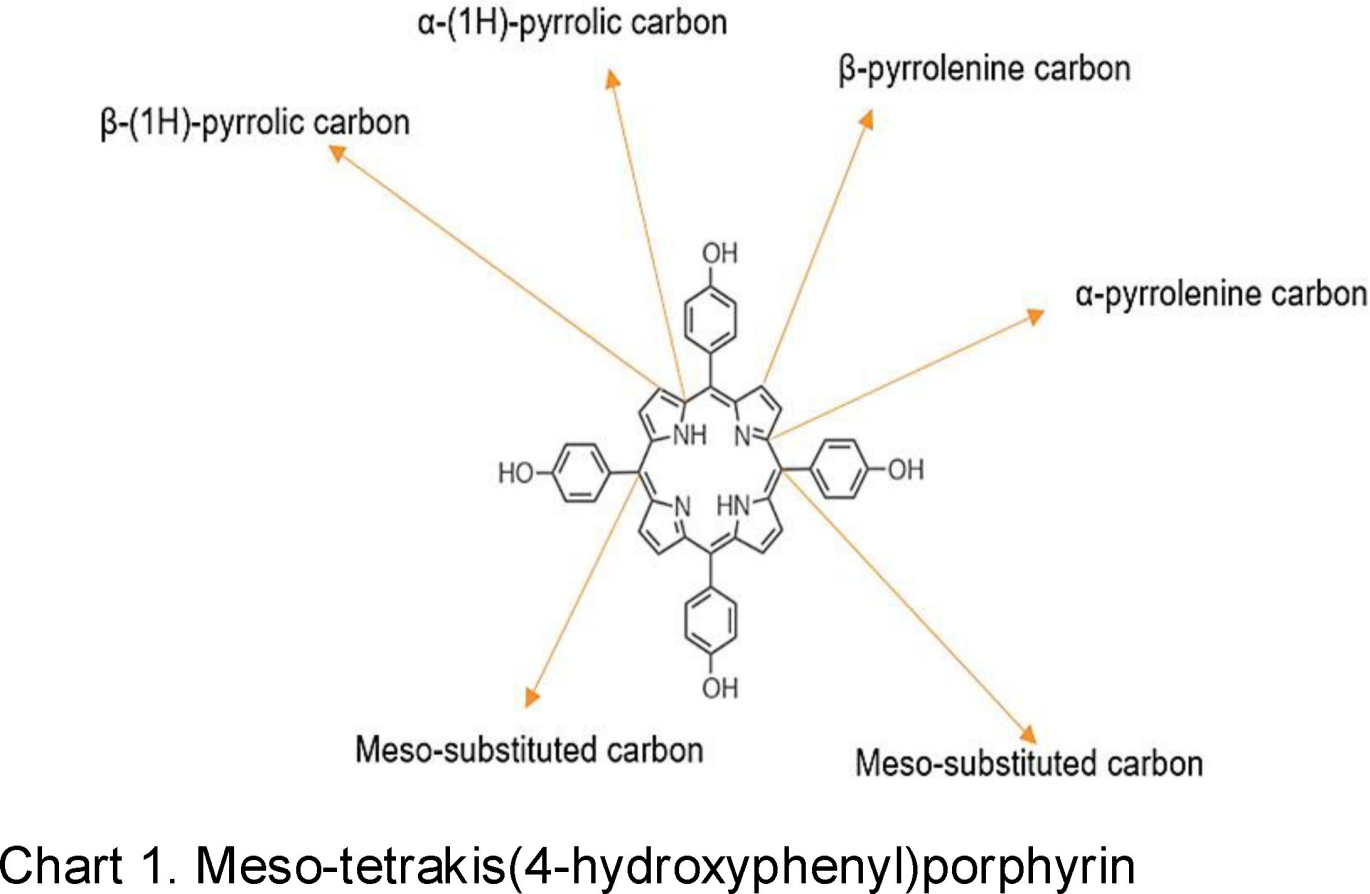
Meso‐tetrakis(4‐hydroxyphenyl)porphyrin

### Morphology of the Porphyrin

**Figure 7:**
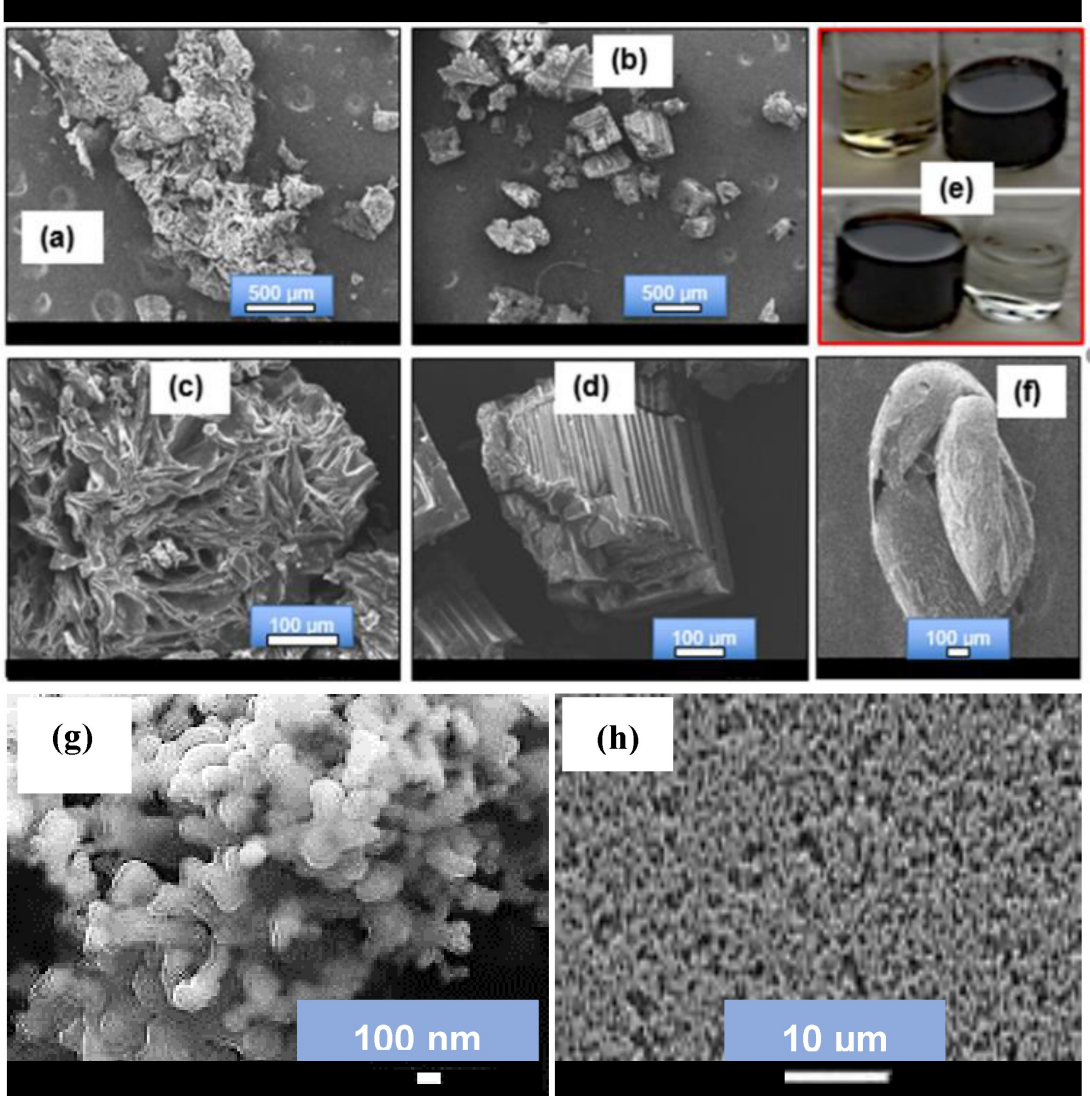
SEM micrographs of the as‐synthesized porphyrin.(a) Porphyrin from AgNDBL electrode; (b) Porphyrin from Au electrode; (c) Higher magnification of (a); (d) Higher magnification of (b); (e) Electrolyzed pyrrole solution (yellow) and its corresponding porphyrin solution (purple) synthesized using AgNDBL electrode (upper images) and electrolyzed pyrrole solution (colourless) and its corresponding porphyrin solution (purple) synthesized using Au electrode; (f) SEM image of a non‐electrolyzed pyrrole solution; (g–h) SEM images of Ag‐NDBL electrode.

The results of the morphology evaluation of the free base porphyrin produced using the electrolyzed pyrrole obtained by cycling across the silver nanodumbbell (AgNDBL) particle and gold (Au) electrode surfaces (for comparison) are given in Figure 7a–d. As shown in Figure 7a–d, the porphyrin evolved as a particulate planar sheet with breakable edges for AgNDBL pyrrole. In contrast, the porphyrin produced by the conventional Au electrode exhibited a layer‐by‐layer sky‐scraper‐like structure. This suggested the supramolecular morphology of the porphyrin can be influenced by the nature of the electrode used for cycling the pyrrole solution (Figure 4i). Figure 7e shows the electrolyzed pyrrole solutions (yellow for AgNDBL and colourless for Au electrodes) and their corresponding purple porphyrin solutions after interaction with the acidified 4‐hydroxybenzaldehyde at room temperature.

**Scheme 2:**
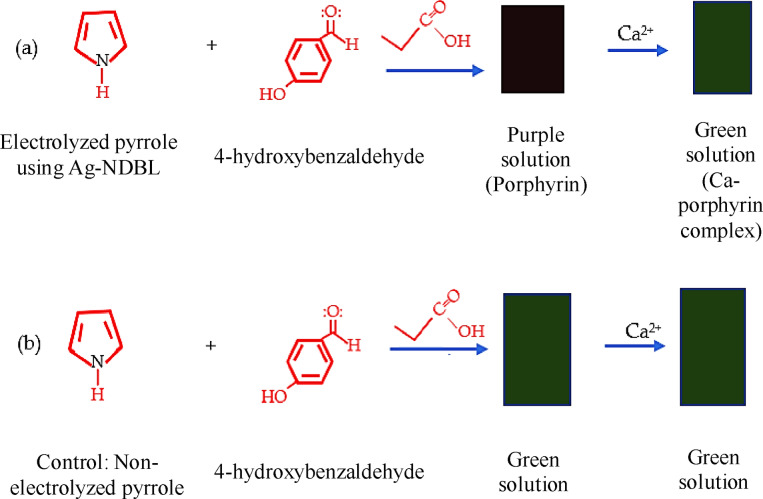
Comparative information between the control and electrolyzed pyrrole solution towards the formation of the targeted porphyrin.

**Figure 8:**
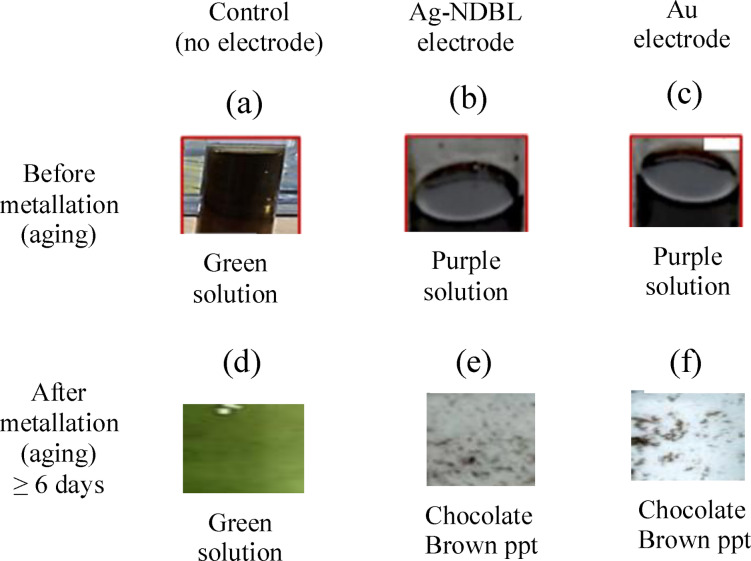
Comparative colour of the control and as‐synthesized porphyrin before and after metalation and aging with Ca^2+^. (a) Non‐electrolyzed pyrrole+4‐hydroxybenzaldehyde+propionic acid; (b) Electrolyzed pyrrole+4‐hydroxybenzaldehyde+propionic acid (using silver nanodumbbell electrode); (c) Electrolyzed pyrrole+4‐hydroxybenzaldehyde+propionic acid (using gold electrode); (d) Non‐electrolyzed pyrrole solution+4‐hydroxybenzaldehyde+propionic acid+Ca^2+^ ion; (e) Electrolyzed pyrrole solution (using silver nanodumbbell electrode)+4‐hydroxybenzaldehyde+propionic acid+Ca^2+^ ion; (f) Electrolyzed pyrrole solution (using gold electrode)+4‐hydroxybenzaldehyde+propionic acid+Ca^2+^ ion.

On the other hand, the control solution, i. e. the non‐electrolyzed pyrrole mixed with acidified 4‐hydroxybenzaldehyde, exhibited different appearance and optical properties. For example, it appeared as a green solution before and after metalation with Ca^2+^ ions, suggesting the formation of a different compound (Scheme 2a–b, Figure 8). Also, after leaving the solution to age for 6 days, no significant precipitation was observed compared to the solution of the targeted porphyrin produced using silver nanodumbbell electrode, which precipitated as a dark chocolate brown solid (Figure 8).

**Figure 9:**
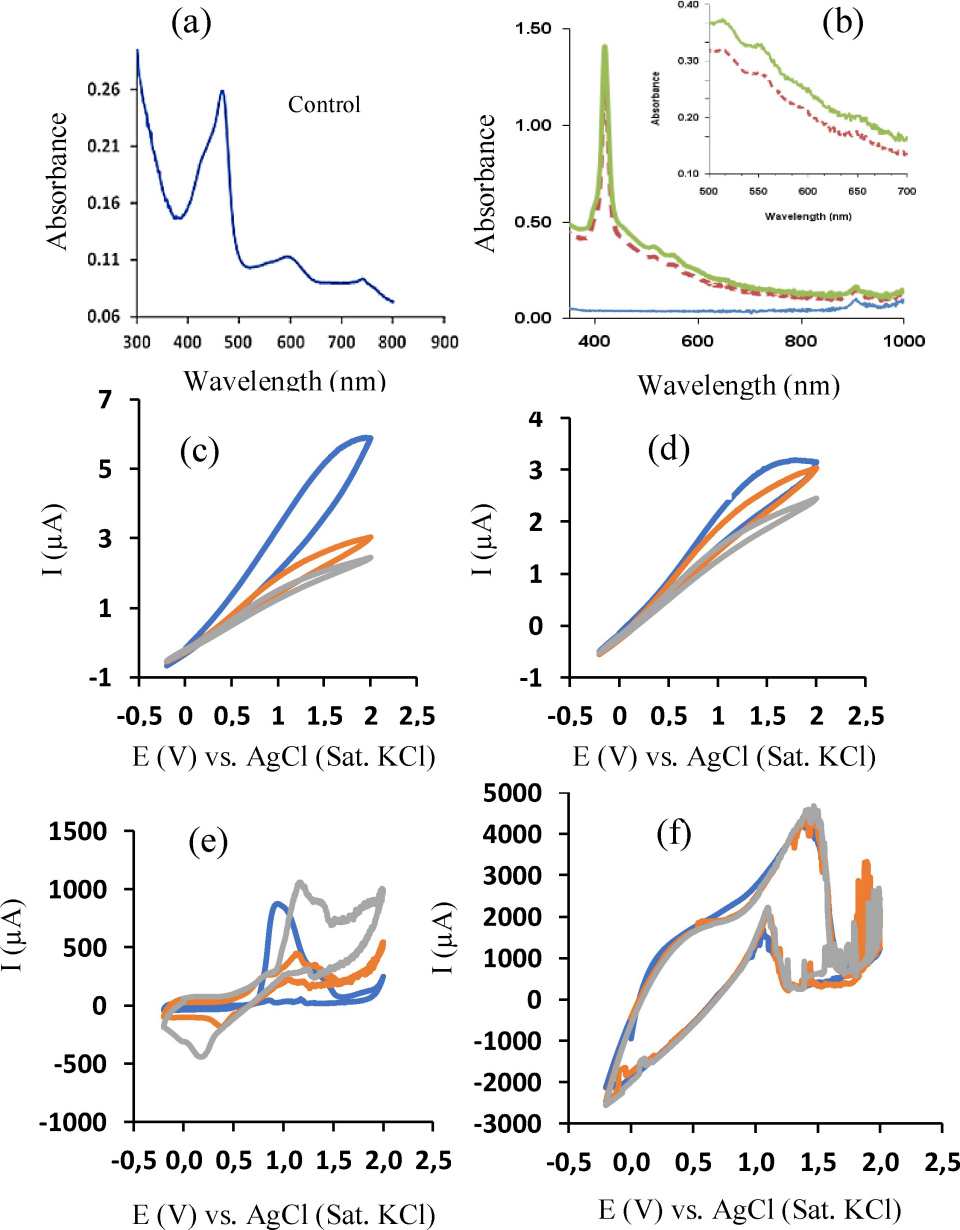
Comparative optical and electrochemical properties. (a) UV‐Vis spectrum of the control solution (non‐electrolyzed pyrrole+4‐hydroxybenzaldehyde+propionic acid (no electrode); (b) UV‐Vis spectrum of electrolyzed pyrrole+4‐hydroxybenzaldehyde+propionic acid (using silver nanodumbbell electrode); (c–d) Cyclic voltammograms of pyrrole solution using silver nanodumbbell electrode; (e–f) Cyclic voltammograms of pyrrole solution using gold electrode.

The optical properties of the control were also different from those of the targeted porphyrin, as shown in Figure 9a–b. According to Figure 9a–b, three peaks at 470, 598 and 749 nm were observed for the control UV‐Vis spectrum, which explained the green colour exhibited by the solution (Scheme 2b). The targeted porphyrin typically has absorption peaks between 419–430 (one Soret band peak) and 500–660 nm (four Q band peaks) (Table 3), which explains its purple colour characteristics. This indicates the importance of electrolyzing the pyrrole solution before mixing it with the acidified aldehyde for porphyrin synthesis.

### Mechanism of Synthesis – The Role of the electrochemical cycling

Cycling the pyrrole solution within a suitable potential window transforms the pyrrole to its oxidized form with the possibility of polymerization occurring, forming higher molecular weight polypyrrole. Termination of the polymerization process may occur after a long cycling causing excess oxidized pyrrole to remain in the electrolyzed solution. Our previous studies using gold electrode revealed cycling the pyrrole solution within the potential window of 0.0 to 2.0 V at 25 mV/s produced quantitatively polypyrrole (mass α 1/no. of cycle) which adsorbed on the surface of the electrode within 16 s of cyclic voltammetric runs.^[29]^ The electrode was used as is (i. e. with the polypyrrole hanging on it) to complete the cycling cycles (35 cycles) before mixing the electrolyzed solution with the acidified 4‐hydroxybenzaldehyde to form the targeted porphyrin. Thus, the electro‐polymerization may play a role in contributing to the effective cyclization (ring formation) of the pyrrole molecules under this condition.

However, using silver nanodumbbell powder electrode, no such polymerization process was visible (no black substance was seen hanging on the electrode). However, a small light brown colour was seen on the surface of the electrode after cycling. This substance was taken to be the adsorbed pyrrole, not polypyrrole since the appearance differed from that of the polypyrrole obtained from the gold electrode under similar conditions. Other information includes the absence of evolution of gas at the counter electrode during the cycling and the pH remaining almost constant across the silver nanodumbbell surface compared to that of the gold.^[29]^ Another unique observation between the gold and silver nanodumbbell particle electrodes was the characteristic difference in their cyclic voltammograms (Figure 9c–f). As shown in Figure 9c–f, while the Au electrode exhibited a voltammogram typically associated with polymerization, the silver nanodumbbell electrode showed a voltammogram more associated with electrocatalytic interactions.^[31]^ Also, while the faradaic oxidation current of the silver nano‐electrode was decreasing with increasing no. of cycle, that of the Au electrode was increasing. Thus, a different explanation will be needed for the synthesis involving the silver nanodumbbell particles.

**Scheme 3:**
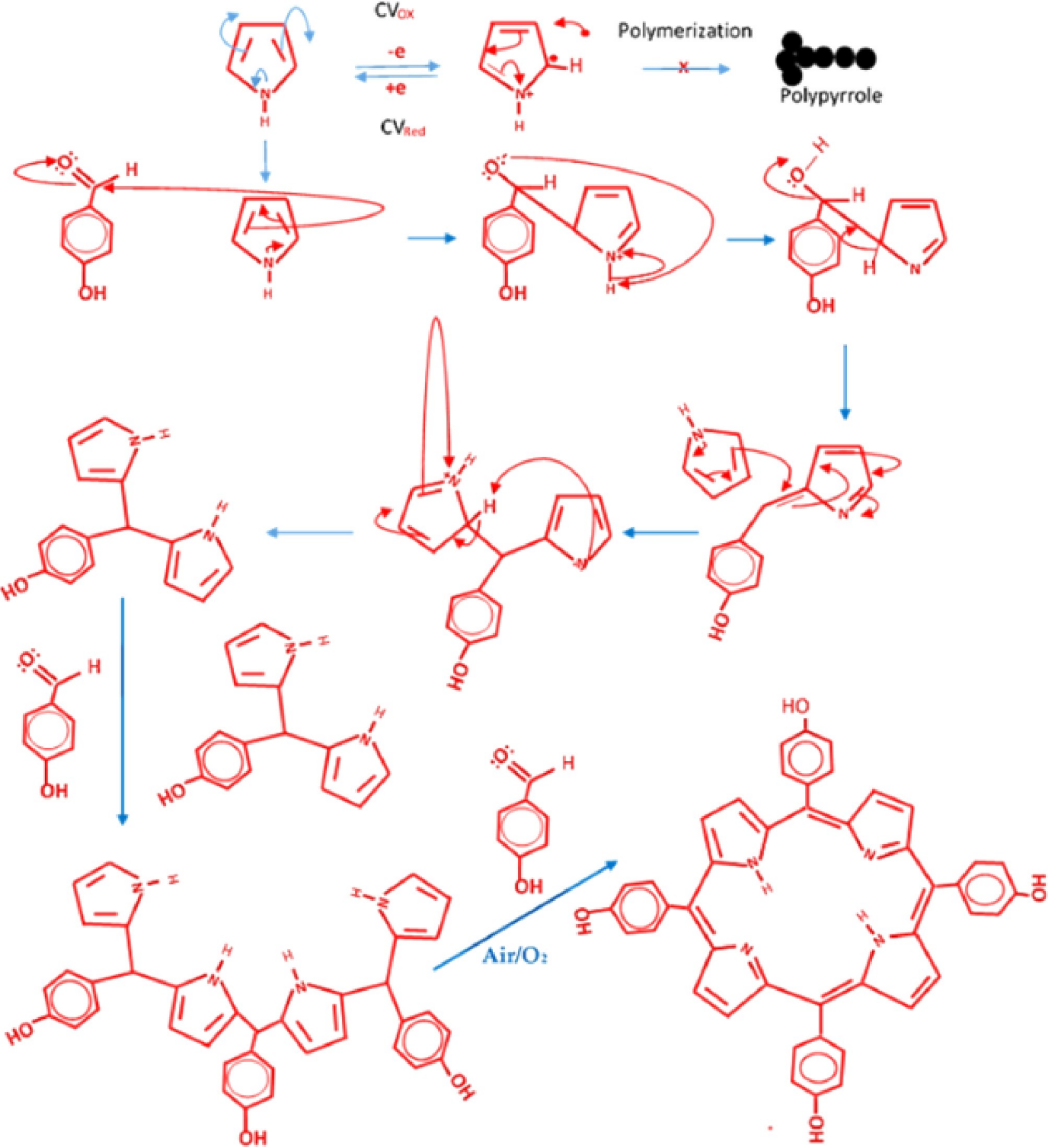
Proposed mechanism of the formation of meso‐tetra(4‐hydroxyphenyl)porphyrin using electrolyzed pyrrole solution.

Nonetheless, since both electrodes produce porphyrins of similar characteristics relative to the targeted porphyrin, it was taken that the polymerization was just a side reaction, probably due to the faster electron exchange on the Au surface. This explains the reason behind the reduction in the absorbance of the pyrrole solution after cycling on the Au surface compared to that of the AgNPs’ surface (Figure 4i). As a result, the following mechanism of synthesis is proposed. First, the cycling of the pyrrole solution between the potential windows of 0.0 and 2.0 V caused the pyrrole to convert to its radical cationic oxidized form while the reverse scan caused a reduction to take place, reducing the oxidized form back to its native neutral form. Due to the slower electron exchange process on the surface of the silver nano‐dumbbell electrode compared to that of the Au‐plated electrode (Figure 3), polymerization cannot be induced. Instead, an adsorptive redox reaction dominated the electrode process. This continuous back‐and‐forth cycling redox process may contribute to the purification of the pyrrole (one of the requirements of the classical synthesis of porphyrin). The lack of gas evolution as well as nearly constant medium pH during and after the cycling also support the possibility of the polymerization of pyrrole not occurring since deprotonation is a well‐established process during polypyrrole formation.[^32^, ^33^] After electrolysis, the pyrrole condensed readily with the acidified 4‐hydroxybenzaldehyde, producing the targeted meso‐tetrakis(4‐hydroxyphenyl)porphyrin (Scheme 3).

## Conclusions

Photoactive visible and near‐infrared absorbing porphyrin and corresponding Ca (II) complex have been successfully synthesized through electrocycling of aqueous pyrrole solution on quasi‐reversible silver nanodumbbell particles’ surface. No requirement for the distillation of the pyrrole solution was needed for this process. Compared to the conventional Au electrode, many differences such as variation in the colour of the electrolyzed solution, cyclic voltammogram and morphology of the evolved porphyrin were observed. The study shows the advantage of using low‐cost powder nanoparticles as electrode materials for the synthesis of symmetrical meso‐substituted supramolecular porphyrins.

**Supporting Information**: Methods and material information attached.

## Conflict of interests

The authors declare no conflict of interest. The funders had no role in the design of the study; in the collection, analyses, or interpretation of data; in the writing of the manuscript; or in the decision to publish the results”.

1

## Supporting information

As a service to our authors and readers, this journal provides supporting information supplied by the authors. Such materials are peer reviewed and may be re‐organized for online delivery, but are not copy‐edited or typeset. Technical support issues arising from supporting information (other than missing files) should be addressed to the authors.

Supporting Information

## Data Availability

The data that support the findings of this study are available from the corresponding author upon reasonable request.
