# Supplementary material for: Electrocatalytic oxidation of pyrrole on a quasi‐reversible silver nanodumbbell particle surface for supramolecular porphyrin production
Source: ChemistryOpen. 2024 Feb 13;13(7):e202300212. doi: 10.1002/open.202300212 (PMC11230922; doi:10.1002/open.202300212)
Supplement: Supplementary file 1 — Supporting Information [file OPEN-13-e202300212-s001.pdf]

ChemistryOpen

Supporting Information

Electrocatalytic oxidation of pyrrole on a quasi-reversible silver nanodumbbell particle surface for supramolecular porphyrin production

Olayemi Jola Fakayode,* Reagan L. Mohlala,* Rudzani Ratshiedana, Bambesiwe M. May, Eno E. Ebenso, Usisipho Feleni, and Thabo T.I. Nkambule

Supplementary Information

Electrocatalytic oxidation of pyrrole on a quasi-reversible silver nanodumbbell particle surface for supramolecular porphyrin production

Olayemi Jola Fakayode[a*], Reagan L. Mohlala[b*], Rudzani Ratshiedana[a], Bambesiwe M. May[b], Eno E. Ebenso[c], Usisipho Feleni[a] and Thabo T.I. Nkambule[a]

O.J. Fakayode, R. Ratshiedana, U. Feleni and T.T.I. Nkambule

Institute for Nanotechnology and Water Sustainability (iNanoWS), College of Science, Engineering and Technology, University of South Africa, Florida Campus, 28 Pioneer, Avenue, Roodepoort, 1709 Johannesburg, South Africa. Emails: olayemifakayode@gmail.com, muthivhirudzani@gmail.com,felenu@unisa.ac.za, nkambtt@unisa.ac.za.

[b] R.L. Mohlala, B.M May:

Advanced Material Science Division, Mintek, 200 Malibongwe Drive, Randburg, Johannesburg, South Africa, Emails: reaganm@mintek.co.za, mbesimay@gmail.com.

[c] Eno E. Ebenso: Centre for Materials Science, College of Science, Engineering and Technology, University of South Africa, Florida Campus, 28 Pioneer Avenue, Roodepoort, 1709, Johannesburg, South Africa, Email: ebensee@unisa.ac.za.

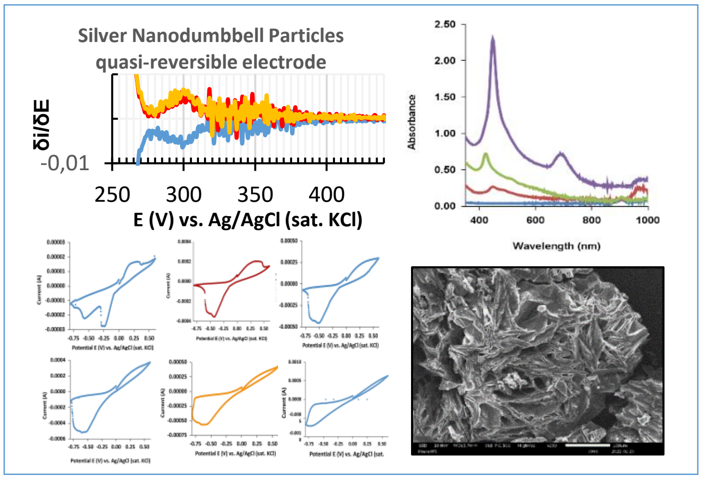

*Corresponding Author's email: olayemifakayode@gmail.com; reaganm@mintek.co.za.

Experimental

All materials were analytical grade (Sigma-Aldrich) and used as received. $CaCl_2$ was obtained from Rochelle Chemicals, South Africa. Ultrapure water (Milli-Q 18.4 Ω.cm) was used to prepare all aqueous solutions. Pyrrole electrolysis was achieved using a three-electrode cell consisting of a silver nanodumbbell particles working electrode, platinum wire counter electrode and silver/silver chloride saturated potassium chloride reference electrode (Ag/AgCl, sat. KCl) connected to an electrochemical workstation (Autolab, Netherland). The solution was run without stirring at room temperature.

Synthesis, purification and yield of materials

Details of the production of the silver nanodumbbell particles were described in our previous report [20]. A three-electrode cell consisting of silver nano-dumbbell powder (working), platinum wire (counter) and silver-silver chloride (saturated KCl) (reference) in contact with an aqueous pyrrole solution in the presence of 0.1 M KCl was employed for the electrochemical cycling under stationary potentiometric mode. Data acquisition was achieved using Metrohm Autolab NOVA 2.0 Software compatible with an AUTOLAB workstation (Netherlands). Briefly, an aqueous pyrrole solution (55 mL, 0.05755 mol/L) was subjected to cyclic voltammetric runs in the presence of 80 mL of 0.1 M KCl solution. The application was programmed to run continuously for 5 cycles before stopping. This process was repeated seven times. The electrolyzed pyrrole solution (3 mL) was condensed with a propionic acid-containing 4-hydroxybenzaldehyde (3 mL, 0.04794 mol/L) by leaving the mixture overnight, forming a purple porphyrin at room temperature. The latter was dried in air in a fume hood, dissolved in absolute ethanol, and crystallized twice in the hood. The yield of the as-synthesized porphyrin was found to be 20.75 % using the relation:

% yield = (Actual mass / Theoretical mass) x 100%

For cyanide interaction, 40 mL of 0.1 M KCl and 40 mL of potassium ferro-cyanide ($[Fe(CN\text{-})_6]^{4-}$) were subjected to cyclic voltammetric runs (1 cycle per scan, changing the scan rate after each cycling stop. The scan rate was varied from 1 mV/s to 1000 mV/s within a potential window of +1 to -1 starting from 0.0 V.

Characterization

The absorption and photoluminescence spectra were obtained using the Shimadzu ultraviolet-visible spectrophotometer and Shimadzu RF-6000 spectrofluorophotometer (Japan), respectively. The morphology of the material was elucidated using scanning electron microscopy (SEM) equipped with an energy-dispersive X-ray probe (Oxford, United Kingdom).

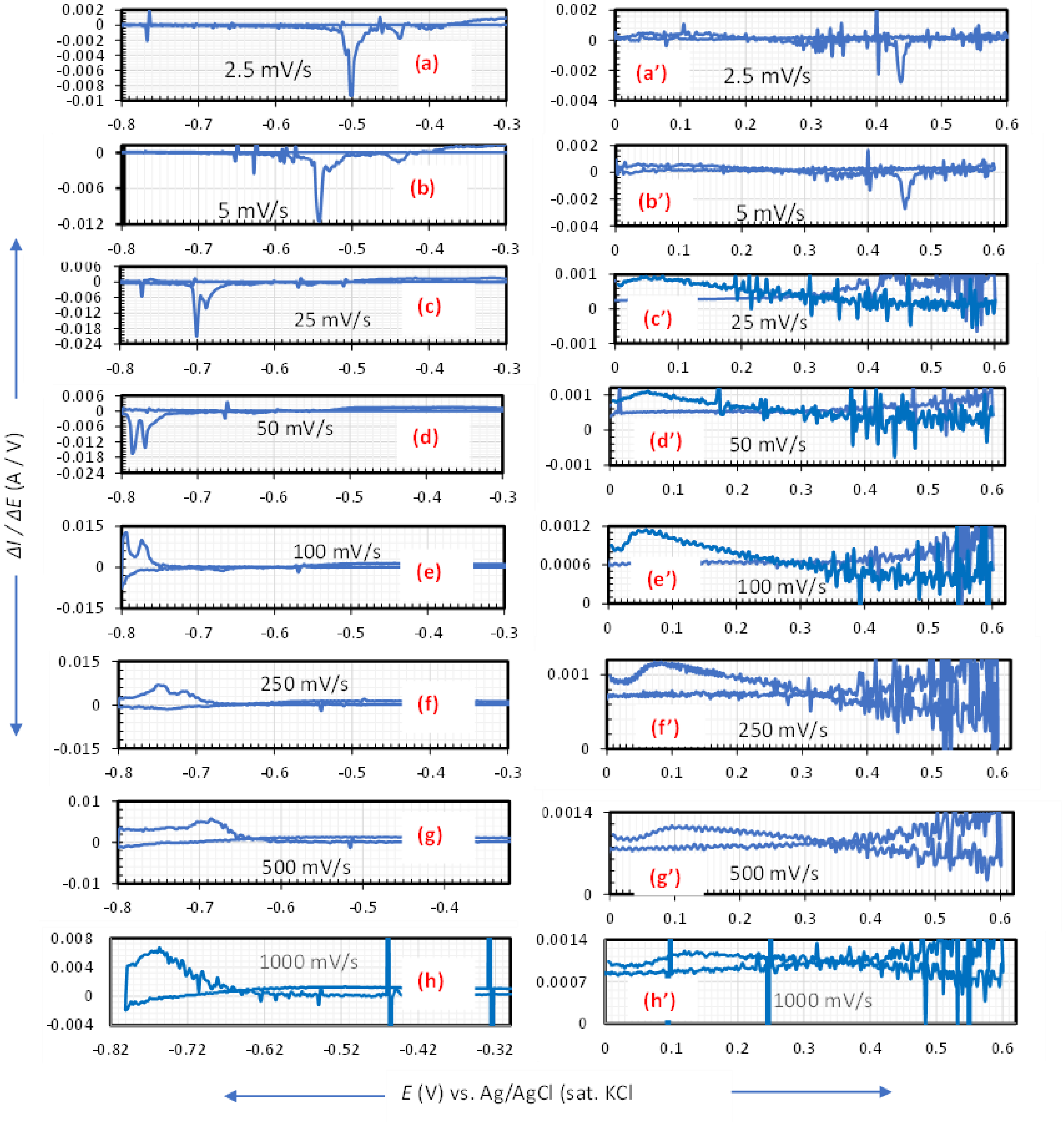

Figure S1. First derivative plots of the voltammograms of AgNDBL at different scan rates in the presence of ferro-cyanide redox probe. (a,a’) 2.5 mV/s; (b,b’) 5 mV/s; (c,c’) 25 mV/s; (d,d’) 50 mV/s; (e,e’) 100 mV/s); (f,f’) 250 mV/s; (g,g’) 500 mV/s; (h,h’) 1000 mV/s. Reference electrode: Ag/AgCl (sat. KCl). Y-axis (δi/δE (A/V), X-axis (E (V)).

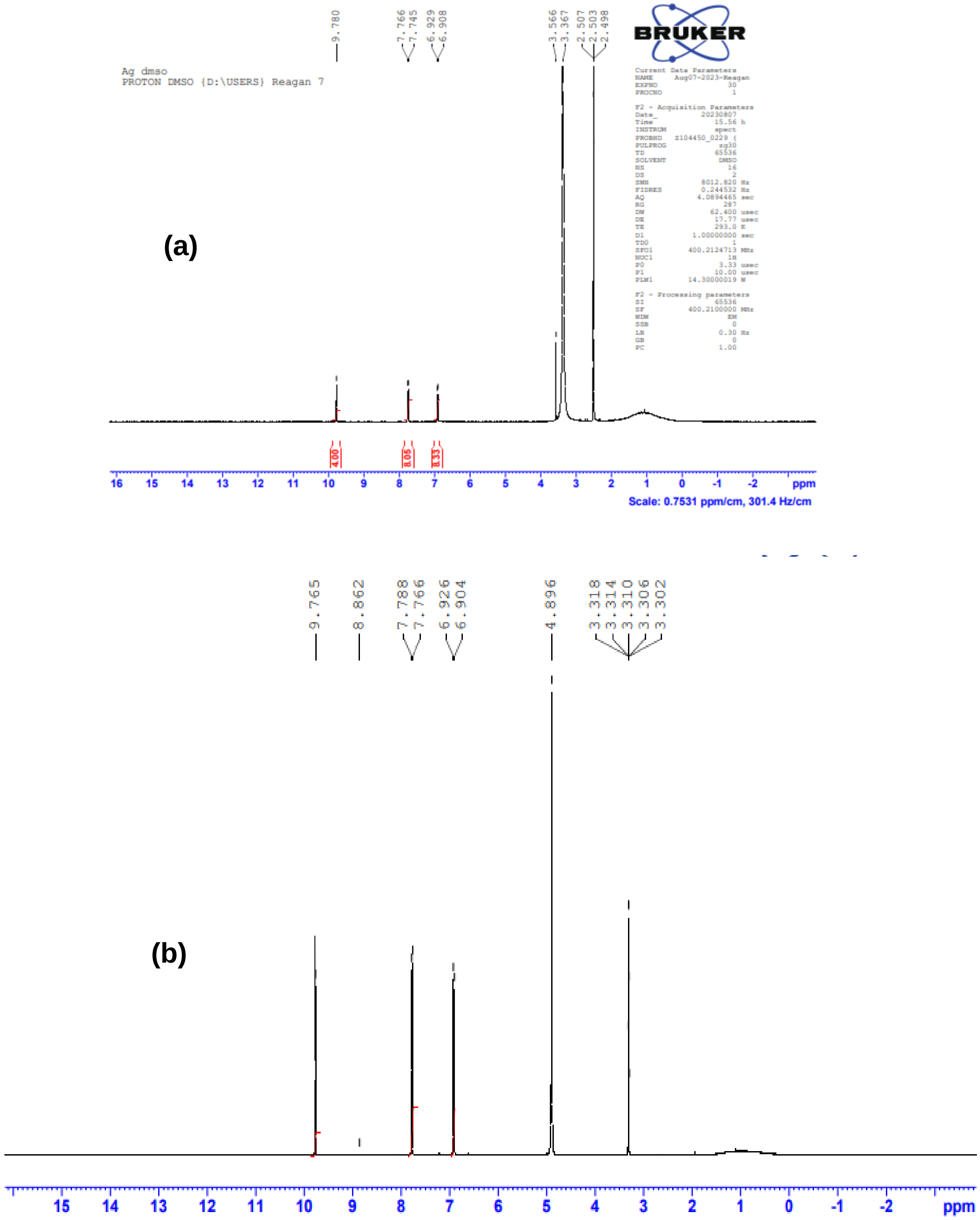

Fig. S2. ^{1}HNMR of the as-synthesized porphyrin.(a) in DMSO-d6; (b) in methanol-d4

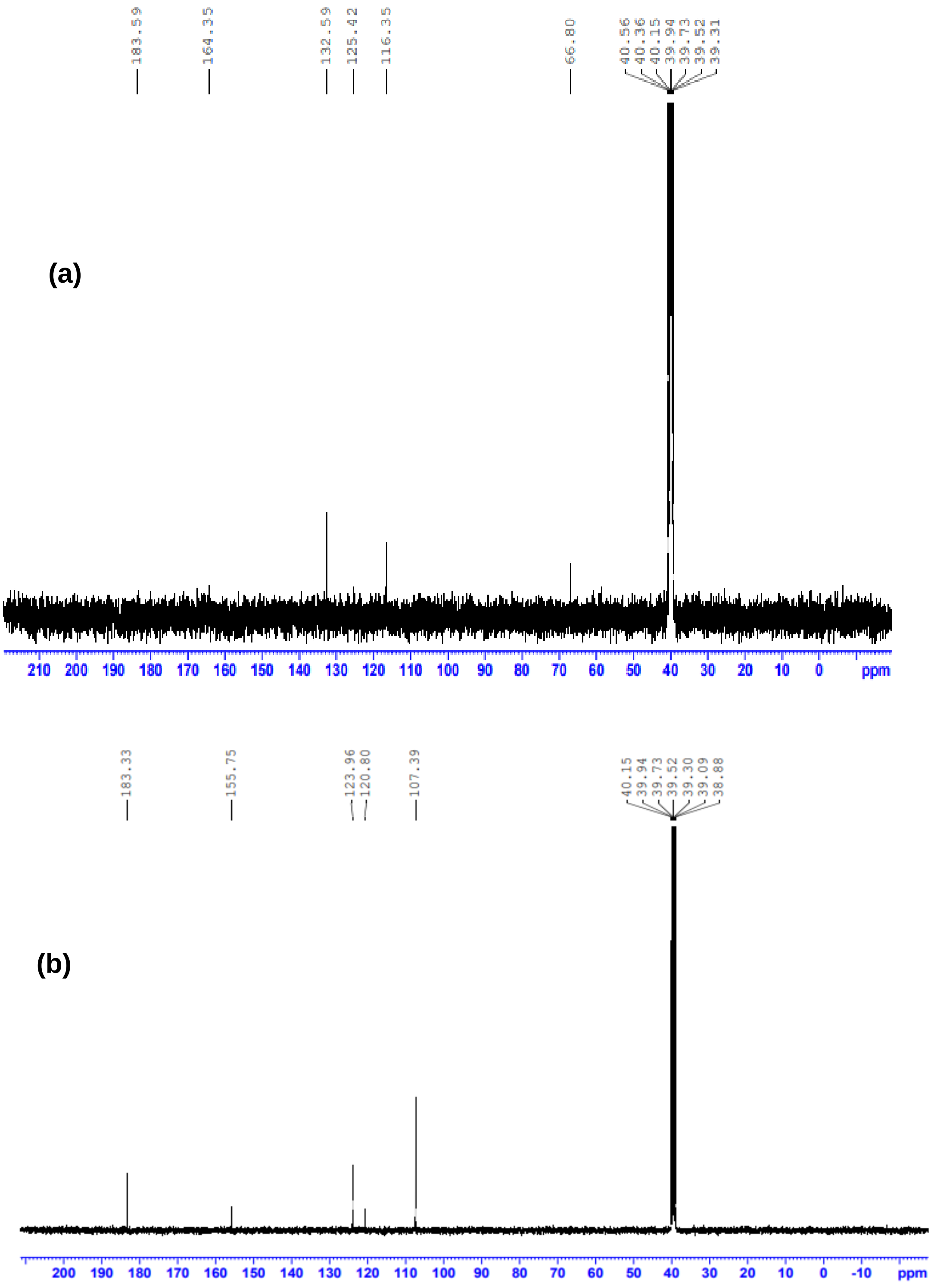

Fig. S3. ^{13}CNMR of the as-synthesized porphyrin. .(a) in DMSO-d6; (b) in methanol-d4.
